# Evolutionary Dynamics of Glycoside Hydrolase Family 1 Provide Insights into Insect–Plant Interactions in Lepidoptera

**DOI:** 10.3390/insects16070727

**Published:** 2025-07-17

**Authors:** Yanping Yuan, Xidan Zhang, Jinyu Wu, Jun Li, Zhengbo He, Wenbo Fu, Amrita Chakraborty, Shulin He

**Affiliations:** 1 College of Life Sciences, Chongqing Normal University, Chongqing 401331, China; yanpingyuan06@126.com (Y.Y.); xidan_zhang@139.com (X.Z.); jinyuwu524@163.com (J.W.); zhengbohe@cqnu.edu.cn (Z.H.); fuice@126.com (W.F.); 2 Chongqing Key Laboratory of Vector Control and Utilization, Institute of Entomology and Molecular Biology, Chongqing Normal University, Chongqing 401331, China; 3 College of Environment and Ecology, Chongqing University, Shabeijie Str. 83, Chongqing 400045, China; jun.li@cqu.edu.cn; 4 Faculty of Forestry and Wood Sciences, Czech University of Life Sciences Prague, Kamýcká 129, 165 00 Prague, Czech Republic; chakraborty@fld.czu.cz

**Keywords:** Lepidoptera insects, glycoside hydrolase family 1 (GH1), β-glucosidase, duplication and loss, gene evolution

## Abstract

Glycoside hydrolase family 1 (GH1) enzymes are essential for plant cell wall digestion and the detoxification of plant metabolites in insects, yet their evolutionary history in Lepidoptera remains unresolved. This study systematically identified *GH1* genes across 61 Lepidopteran genomes and analyzed their evolutionary dynamics. In addition, the expression profiles of *GH1* genes in the silkworm (*Bombyx mori*) across various developmental stages and tissues were related to their evolutionary histories. A total of 996 *GH1* genes were annotated and classified into 11 groups, with each showing distinct species diversity. Gene duplication and loss analysis revealed frequent duplications and losses during Lepidoptera evolution; these duplications primarily originated through tandem and dispersed duplications and were located in syntenic regions. Transcriptomic analysis of the silkworm revealed that the groups and duplications of *GH1* genes were correlated to their expression patterns, with high expression in the larval midgut and fat body. These findings suggest that *GH1* gene duplications and losses and expression have played a significant role in Lepidopteran adaptation to diverse host plants. Overall, this study provides comprehensive insights into the evolutionary trajectories of *GH1* genes, highlighting their potential contribution to insect–plant interactions in Lepidoptera.

## Simple Summary

Lepidopteran insects rely on special enzymes to break down plant materials and protect themselves from plant toxicants. One important group of these enzymes is glycoside hydrolase family 1 (GH1). In this study, we examined *GH1* genes in 61 species of butterflies and moths to understand how these genes evolved and how they help insects interact with plants. We identified 996 *GH1* genes and grouped them into 11 categories, with each displaying different species diversity. Most *GH1* genes originated through gene duplications, especially tandem and dispersed duplications. In addition, we examined the expression of these genes in the silkworm and found certain highly expressed *GH1* genes during larval stages, especially in tissues involved in digestion. These results showed that the evolutionary history of *GH1* genes in Lepidoptera reflects their adaptation to plant feeding, providing insights for further investigation into plant–insect interactions.

## 1. Introduction

Lepidopteran insects, comprising butterflies and moths, encompass a remarkable diversity of species, with approximately 180,000 described species [1,2]. The adults play essential ecological roles as pollinators, feeding primarily on nectar or pollen, while their larvae are predominantly herbivorous. Most Lepidopteran larvae primarily consume leaves, while some feed on stems, flowers, fruits, or wood. Several species are of major agricultural concern, as their larvae are destructive pests that cause significant damage to agricultural production [3]. These herbivorous larvae rely on a diverse suite of carbohydrate-active enzymes (CAZymes) to digest and metabolize plant cell components. Among these, glycoside hydrolases (GHs), a class of enzymes that hydrolyze glycosidic compounds, are critical in breaking down plant cell wall polysaccharides.

Within the GH class, glycoside hydrolase family 1 (GH1) is a key enzyme that plays a pivotal role in the herbivory of Lepidopteran larvae by mediating the digestion of plant cell walls and detoxification of plant secondary metabolites. GH1 enzymes contribute to the degradation of plant cell wall cellulose by acting synergistically with other cellulases (e.g., endoglucanases and exoglucanases) to convert β-glucans into glucose monomers, thereby facilitating cellulose breakdown [4,5]. For instance, the codling moth *Cydia pomonella* employs GH1 enzymes to degrade hemicellulose released from plant cell walls into monosaccharides, facilitating cellulose digestion [6]. In addition, GH1 enzymes are also involved in the detoxification of phytotoxin precursors by specifically hydrolyzing glycosidic bonds in plant defensive secondary metabolites, such as cyanogenic glycosides and glucosinolates [7]. This enzymatic activity mitigates toxicity and enables larvae to feed on chemically defended plants. A notable example is the cabbage white butterfly *Pieris rapae*, which uses GH1 enzymes to cleave the thioglucosidic bonds of glucosinolates, converting toxic precursors into less harmful nitriles [8]. The integration of these functions not only underscores the dual role of GH1 in energy acquisition and defensive detoxification but also reveals its evolutionary strategy in enabling herbivorous insects to overcome both the physical barriers and chemical defenses of plants.

Despite the functional importance of *GH1*, the evolutionary dynamics of this gene family in Lepidoptera and its potential role in driving ecological adaptation to diverse plant hosts remain poorly understood. In particular, it is unclear how variations in *GH1* gene copy number, duplication mode, and expression patterns contribute to differences in dietary specialization and ecological niche occupation among Lepidopteran lineages.

Glycoside hydrolase family 1 (GH1) exhibits a highly conserved catalytic mechanism across bacteria, insects, vertebrates, and plants [9]. It possesses the canonical (β/α)8 TIM barrel supersecondary structure [10]. Its active site is situated in the loop regions of β-strands within the hydrophobic core, where a catalytic triad (conserved glutamate/aspartate residues) facilitates glycosidic bond cleavage through an acid–base catalytic mechanism [11]. This structural conservation underpins the functional diversity of GH1 [12], while the differential fusion of auxiliary domains at its termini drives lineage-specific functional expansion in herbivorous insects during adaptation-driven evolution [13].

This study employed a comprehensive analysis of the *GH1* gene family in 61 Lepidoptera genomes, representing a broad taxonomic and ecological spectrum, to investigate the adaptive evolution of the *GH1* gene family in insect–plant interactions. After exhaustively annotating the *GH1* genes, we explored their evolutionary trajectories by inferring their phylogeny and duplication histories, as well as analyzing their duplication patterns and collinearity. We further integrated transcriptomic data across developmental stages and tissues of the silkworm (*Bombyx mori*) to assess the role of *GH1* in mediating the intricate Lepidoptera–plant interactions, providing novel insights into the coevolutionary dynamics between insects and their host plants.

## 2. Materials and Methods

### 2.1. Data Collection

In this study, we downloaded genome data for 61 Lepidoptera species (representing a broad taxonomic and ecological spectrum) from the NCBI database (Table 1). The quality of genome assemblies was assessed using BUSCO v5.4.3 and the insecta_odb10 database [14], with genomic data filtered based on a completeness threshold of 95%. A transcriptomic dataset for the silkworm (*B. mori*) was obtained from the NCBI SRA database (BioProject ID: PRJNA559726). This dataset encompasses transcriptomes from ten developmental stages (4th instar day 3, fourth larval molting, 5th instar day 0, 5th instar day 3, wandering, pre-pupa, pupa day 1, pupa day 4, pupa day 7–8, and moth day 1) and 16 body tissues (anterior silk gland, middle silk gland, posterior silk gland, testis, ovary, midgut, fat body, malpighian tubules, hemolymph, trachea, epidermis, head, thorax, antennae, legs, and wings) [15].

### 2.2. Genome-Wide Identification of GH1

The *GH1* family genes were identified from genomic data in the RefSeq and GenBank databases using distinct strategies. For RefSeq data, the longest transcript isoform of each gene was first extracted as the representative sequence using the R package orthologr [16]. Subsequently, the protein sequences encoded by these transcripts were subjected to GH1 prediction using the run_dbcan tool v4.14. This tool integrates three methods—HMMER, Diamond, and dbCAN_sub [17]—and only genes encoding proteins with consistent predictions across all three methods were classified as *GH1* family members.

For GenBank data, we employed a combined approach of homology prediction and run_dbcan analysis to identify *GH1* genes. Firstly, CD-HIT v4.8.1 [18] was used to cluster GH1 protein sequences predicted from RefSeq genomes with a sequence identity threshold of 0.95 and a word size of 5, constructing a non-redundant GH1 database. Subsequently, genblastG was utilized to perform homology alignment between GenBank genomic data and the non-redundant GH1 database to identify potential *GH1* genes. CDS sequences lacking start or stop codons were filtered using GffRead v0.12.7 [19], and redundant sequences were removed. For *GH1* gene models with overlapping genomic positions, the model with the highest genblastG score was selected as the final candidate. Finally, following the same annotation pipeline as applied to RefSeq data, run_dbcan was used to further validate the protein sequences of the filtered *GH1* gene models. Only gene models encoding proteins annotated as *GH1* through this pipeline were retained as high-confidence *GH1* genes.

### 2.3. Gene Tree Inference

To infer the phylogenetic relationships of *GH1* genes in Lepidoptera, we aligned the predicted GH1 protein sequences using MAFFT v7.520 [20] and MUSCLE v3.8.1551 [21]. The alignment was subsequently refined with rascal v1.34 [22], and both the raw and refined alignments were scored using normd v1.2. The highest-scoring alignment was selected for phylogenetic tree construction via the maximum likelihood method implemented in IQ-TREE v2.2.0 [23] with 1000 ultrafast bootstrap replicates and the NQ insect substitution model. Subsequently, we categorized the *GH1* genes into groups by using Possvm v1.2 with default settings, which uses species overlap and Markov clustering algorithms to cluster the gene families into orthology clusters [24]. Finally, the phylogenetic tree was visualized using iTOL v7 [25].

### 2.4. Gene Duplication and Loss Inference

To analyze changes in *GH1* gene number during the evolutionary history of Lepidoptera, we reconciled the *GH1* gene tree with a published species tree [26] using Notung v2.9 [27] to infer gene duplication and loss events. Parameters for Notung were set as the following default settings: loss cost = 1.0; duplication cost = 1.5; transfer cost = 3.0; co-divergence cost = 0; and edge weight threshold = 90% of the maximum edge weight.

### 2.5. Duplicate Mode Inference and Collinearity Analysis

Seven representative species, spanning the main Lepidopteran families, with chromosomal level genome assemblies from the RefSeq database were selected for analysis, including *B. mori*, *Hyposmocoma kahamanoa*, *Ostrinia furnacalis*, *Pectinophora gossypiella*, *Bicyclus anynana*, *Papilio machaon*, and *Colias croceus*. The duplication modes of *GH1* genes in these species were inferred using the duplicate_gene_classifier tool in MCScanX v1.0.0 [28]. Collinearity analysis was subsequently performed with MCScanX, and Circos plots were generated using TBtools v2.119 [29] to visualize genomic collinearity.

### 2.6. Expression of GH1 Genes in Developmental Stages and Tissues of B. mori

Raw transcriptomic data of *B. mori* were subjected to quality control and filtering using Fastp v0.23.4 [30] to obtain high-quality reads. These reads were aligned to the reference genome analyzed in this study using HISAT2 v2.2.1 [31]. Gene quantification was performed using FeatureCounts v2.0.6 [32] to generate a gene expression matrix. Raw expression counts were normalized as Transcripts Per Million (TPM) values, followed by log2 (TPM + 1) transformation. Finally, the expression profiles of target genes were visualized using the pheatmap v 1.0.12 R package.

**Table 1:** The number of GH1 genes in Lepidoptera species.

Superfamily	Family	Genus	Species	Accession No.	GH1	Feeding Habit
Bombycoidea	Bombycidae	Bombyx	mori	GCF_014905235.1	20	M
Bombycoidea	Bombycidae	Trilocha	varians	GCA_030269945.2	18	P
Bombycoidea	Lasiocampidae	Dendrolimus	pini	GCA_949752895.1	10	P
Copromorphoidea	Carposinidae	Carposina	sasakii	GCA_014607495.2	10	P
Cossoidea	Cossidae	Zeuzera	pyrina	GCA_907165235.1	9	P
Gelechioidea	Coleophoridae	Coleophora	deauratella	GCA_958295455.1	10	M
Gelechioidea	Coleophoridae	Coleophora	flavipennella	GCA_947284805.1	13	
Gelechioidea	Cosmopterigidae	Hyposmocoma	kahamanoa	GCF_003589595.1	15	
Gelechioidea	Gelechiidae	Anarsia	innoxiella	GCA_947563765.1	18	
Gelechioidea	Gelechiidae	Athrips	mouffetella	GCA_947532105.1	14	M
Gelechioidea	Gelechiidae	Carpatolechia	fugitivella	GCA_951230895.1	8	P
Gelechioidea	Gelechiidae	Pectinophora	gossypiella	GCF_024362695.1	10	P
Gelechioidea	Gelechiidae	Scrobipalpa	costella	GCA_949820665.1	10	M
Geometroidea	Geometridae	Biston	stratarius	GCA_950106695.1	12	
Geometroidea	Geometridae	Eulithis	testata	GCA_947507515.1	11	*P*
Geometroidea	Geometridae	Hemistola	chrysoprasaria	GCA_947063395.1	14	M
Geometroidea	Geometridae	Horisme	vitalbata	GCA_951804965.1	9	M
Geometroidea	Geometridae	Lampropteryx	suffumata	GCA_948098915.1	16	M
Hesperioidea	Hesperiidae	Carterocephalus	palaemon	GCA_944567765.1	18	M
Hesperioidea	Hesperiidae	Erynnis	tages	GCA_905147235.1	22	M
Hesperioidea	Hesperiidae	Pyrgus	malvae	GCA_911387765.1	16	M
Hesperioidea	Hesperiidae	Thymelicus	acteon	GCA_951805285.1	22	M
Hesperioidea	Hesperiidae	Thymelicus	sylvestris	GCA_911387775.1	19	M
Incurvarioidea	Adelidae	Nematopogon	swammerdamellus	GCA_946902875.1	13	
Incurvarioidea	Incurvariidae	Incurvaria	masculella	GCA_946894095.1	14	M
Noctuoidea	Erebidae	Euproctis	similis	GCA_905147225.2	11	P
Noctuoidea	Erebidae	Leucoma	salicis	GCA_948253155.1	13	P
Noctuoidea	Erebidae	Lymantria	dispar	GCA_016802235.1	14	P
Noctuoidea	Erebidae	Lymantria	monacha	GCA_905163515.2	17	P
Noctuoidea	Erebidae	Orgyia	antiqua	GCA_916999025.1	13	P
Noctuoidea	Notodontidae	Clostera	curtula	GCA_905475355.2	8	M
Noctuoidea	Notodontidae	Furcula	furcula	GCA_911728495.1	17	P
Noctuoidea	Notodontidae	Notodonta	dromedarius	GCA_905147325.1	14	P
Noctuoidea	Notodontidae	Phalera	bucephala	GCA_905147815.2	9	P
Noctuoidea	Notodontidae	Ptilodon	capucinus	GCA_914767695.1	10	
Papilionoidea	Lycaenidae	Aricia	agestis	GCF_905147365.1	29	P
Papilionoidea	Lycaenidae	Celastrina	argiolus	GCA_905187575.2	13	P
Papilionoidea	Lycaenidae	Cyaniris	semiargus	GCA_905187585.1	18	P
Papilionoidea	Lycaenidae	Lycaena	phlaeas	GCA_905333005.2	21	P
Papilionoidea	Lycaenidae	Polyommatus	icarus	GCA_937595015.1	24	P
Papilionoidea	Nymphalidae	Bicyclus	anynana	GCF_947172395.1	35	M
Papilionoidea	Nymphalidae	Danaus	plexippus	GCF_009731565.1	19	M
Papilionoidea	Nymphalidae	Melitaea	cinxia	GCF_905220565.1	26	M
Papilionoidea	Nymphalidae	Nymphalis	io	GCF_905147045.1	13	P
Papilionoidea	Nymphalidae	Pararge	aegeria	GCF_905163445.1	17	M
Papilionoidea	Papilionidae	Battus	philenor	GCA_028537555.1	18	P
Papilionoidea	Papilionidae	Iphiclides	podalirius	GCA_933534255.1	20	P
Papilionoidea	Papilionidae	Papilio	machaon	GCF_912999745.1	18	M
Papilionoidea	Pieridae	Colias	croceus	GCF_905220415.1	22	M
Papilionoidea	Pieridae	Leptidea	sinapis	GCF_905404315.1	42	M
Papilionoidea	Pieridae	Pieris	rapae	GCF_905147795.1	21	M
Papilionoidea	Pieridae	Pieris	brassicae	GCF_905147105.1	25	P
Papilionoidea	Pieridae	Pieris	napi	GCF_905475465.1	27	M
Pyraloidea	Crambidae	Calamotropha	paludella	GCA_927399485.1	11	M
Pyraloidea	Crambidae	Chilo	suppressalis	GCA_902850365.2	18	M
Pyraloidea	Crambidae	Cnaphalocrocis	medinalis	GCA_014851415.1	12	P
Pyraloidea	Crambidae	Ostrinia	furnacalis	GCF_004193835.3	15	P
Sesioidea	Choreutidae	Choreutis	nemorana	GCA_949316135.1	9	M
Yponomeutoidea	Argyresthiidae	Argyresthia	goedartella	GCA_949825045.1	13	M
Yponomeutoidea	Lyonetiidae	Leucoptera	coffeella	GCA_030578115.1	10	M
Zygaenoidea	Limacodidae	Apoda	limacodes	GCA_946406115.1	23	M

P: polyphagous, M: monophagous. The feeding habits of the Lepidopteran species were defined based on data from the HOSTS database and a previously published study [33]. A species is categorized as polyphagous if it feeds on plants from more than one family.

## 3. Results

### 3.1. Phylogenetic Tree of Lepidoptera

**Figure 1:**
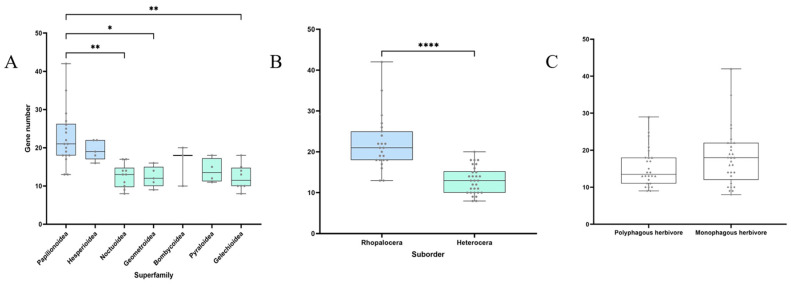
The number of *GH1* genes in each family of Lepidoptera insects (**A**); the number of *GH1* genes in butterflies and moths (**B**); comparison of the number of *GH1* genes between polyphagous herbivores and monophagous herbivores. ▲ represents butterflies, and ◆ represents moths (**C**). Blue and green represent the two Lepidopteran suborders, Rhopalocera and Heterocera, respectively. Non-parametric tests were used to analyze differences among Lepidopteran groups: multiple group comparisons (Kruskal–Wallis test) and pairwise comparisons (Dunn’s test) among 7 superfamilies (**A**), two-group comparison (Mann–Whitney test) between Rhopalocera and Heterocera in (**B**). Asterisks *, **, and **** represent significant differences in *p* < 0.05, 0.01, and 0.0001, respectively.

We identified a total of 996 *GH1* genes across 61 Lepidopteran genomes, with an average of approximately 16 copies per species (Table 1). Among these, Leptidea sinapis exhibited the highest number of *GH1* genes (42), while *Carpatolechia fugitivella* and *Clostera curtula* had the lowest (8 copies each). The number of *GH1* genes varied significantly across superfamilies (Kruskal–Wallis test, *p* < 0.0001). The superfamilies Papilionoidea, Hesperioidea, and Zygaenoidea showed relatively higher average *GH1* gene numbers (~20 copies), with Papilionoidea displaying the highest average gene number (~23 copies; Figure 1) and significantly different from Noctuoidea, Geometroidea, and Gelechioidea (Dunn’s test, adjusted *p* = 0.0012, 0.0276, and 0.0024, respectively). In contrast, the superfamilies Cossoidea, Sesioidea, and Copromorphoidea had low average gene numbers (9–10 copies; Table 1). Notably, within Papilionoidea, *GH1* gene numbers varied substantially among species, ranging from 13 copies in *Celastrina argiolus* to 42 copies in *Leptidea sinapis*. By comparison, other superfamilies exhibited more consistent *GH1* gene counts. In addition, the *GH1* gene numbers between the two suborders, Rhopalocera and Heterocera, were significantly different from each other (Mann–Whitney test, *p* < 0.0001).

**Figure 2:**
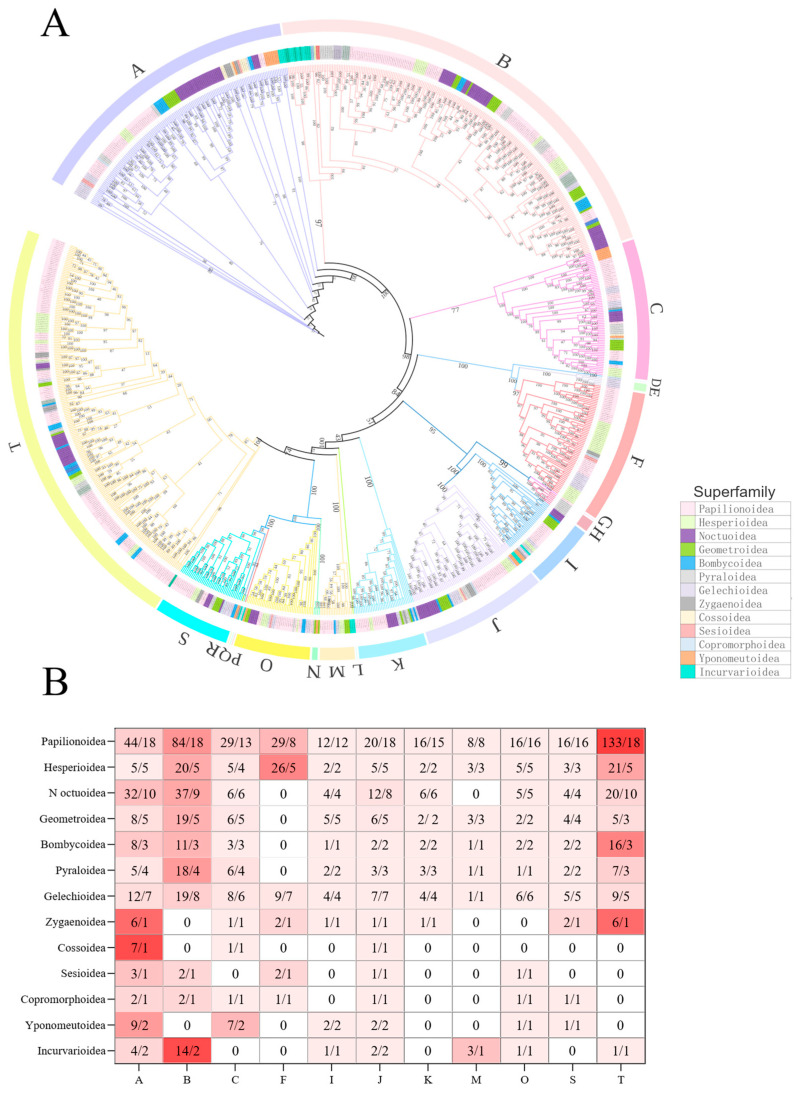
The phylogenetic tree of *GH1* genes in Lepidoptera (**A**); heatmap of the copy number of the *GH1* gene in Lepidoptera insects among different groups and superfamilies (**B**). The phylogenetic tree of *GH1* genes in Lepidoptera was constructed from protein sequences using IQTREE and visualized with iTOL v6. The bootstrap values were labeled at each node. Tip labels include gene IDs and genus names, while branches are color-coded by taxonomic group. The predicted *GH1* genes were divided into 20 groups by using possvm in the phylogenetic tree and then summarized in a heat map representing 11 main groups in Lepidoptera superfamilies (**A**). The numbers in each cell represent the total gene number/the species number of each gene group in each Lepidopteran superfamily. The colors indicate the average gene number of each gene group in superfamilies (**B**).

The phylogenetic tree of *GH1* genes in Lepidoptera (Figure 2A) reveals a complex architecture within this gene family. Based on species overlap and clustering in the gene tree, we classified *GH1* genes into 20 groups (Group A–T) with 11 main groups. The main groups represent the majority of the genes, and the other nine groups only contain 22 *GH1* genes. Groups A and I include members from all Lepidopteran superfamilies (Figure 2B). Groups B, F, and T are predominantly composed of *GH1* genes from butterfly-associated superfamilies. Groups C, I, O, and S also exhibit high diversity, containing *GH1* genes from 10 to 11 superfamilies but lack representatives from certain superfamilies. For instance, Group C lacks Sesioidea and Incurvarioidea, while Group I lacks Sesioidea, Cossoidea, and Copromorphoidea. Groups F, K, and M display low diversity, encompassing GH1 genes from six to eight Lepidopteran superfamilies. Interestingly, over half of Group T is composed of *GH1* genes from the Papilionoidea superfamily. In the minority groups, most of the genes are from moths, except for only one gene from *Danaus plexippus*.

The distribution of *GH1* genes across phylogenetic trees exhibited marked differences among Lepidopteran taxa, with notable incongruence between gene trees and species trees. *GH1* genes from moths were predominantly clustered in Groups A, B, and T, while those from butterflies were distributed more frequently in Groups A, B, F, and T.

Notably, species of the superfamily Papilionoidea were represented in all the main groups. Additionally, species-specific gene distribution patterns were observed, for instance, genes of *Zeuzera pyrina* were sparsely represented in Groups A, C, and J, whereas those of *Choreutis nemorana* occurred in limited numbers across Groups A, B, F, J, and O. These patterns underscore the extensive diversity within the GH1 gene family.

### 3.2. Gene Duplications and Losses

**Figure 3:**
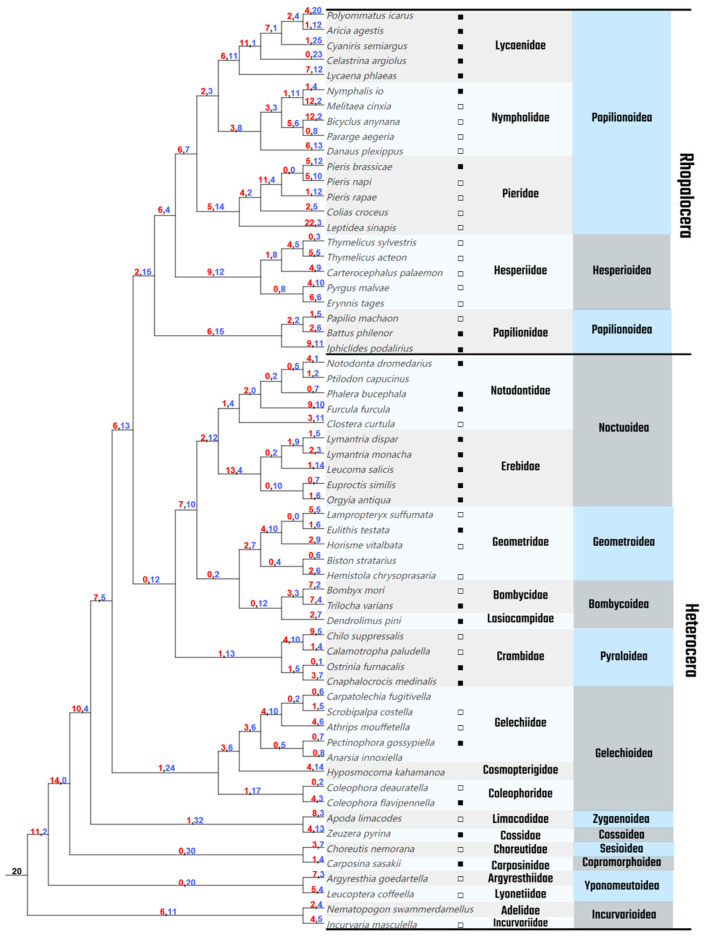
Duplication and loss of *GH1* genes in Lepidoptera. The duplication and loss of *GH1* genes in Lepidoptera were inferred by reconciling the *GH1* gene tree with the species tree; the species tree was derived from a previously published study [26]. Red numbers indicate duplication events, while blue numbers represent gene losses; ■ represents a polyphagous herbivore, and □ represents a monophagous herbivore.

Through evolutionary inference of duplication and loss events in multi-copy gene families across Lepidoptera, we found that the *GH1* gene family has undergone frequent gene duplication and loss during its evolutionary history. The ancestral Lepidopteran lineage likely harbored 20 *GH1* gene copies, with significant duplication expansions occurring near early branching nodes (Figure 3). However, as evolution progressed, *GH1* genes exhibited extensive loss across most internal evolutionary nodes, particularly those closer to terminal branches. In families such as Lyonetiidae, Argyresthiidae, Carposinidae, Choreutidae, Cossidae, and Limacodidae, nearly 30 *GH1* genes were lost within their internal evolutionary nodes. Terminal branches in the Lycaenidae family retained only a limited number of duplications (0–7) but experienced substantial losses (12–25). In contrast, species in the Pieridae family displayed relatively active duplication activity, with internal nodes showing 0–11 duplications and terminal branches reaching up to 22 duplications (*Leptidea sinapis*). Notably, butterfly clades exhibited significantly higher *GH1* duplication rates compared to moth lineages.

### 3.3. Duplication Modes and Collinearity Analysis

**Figure 4:**
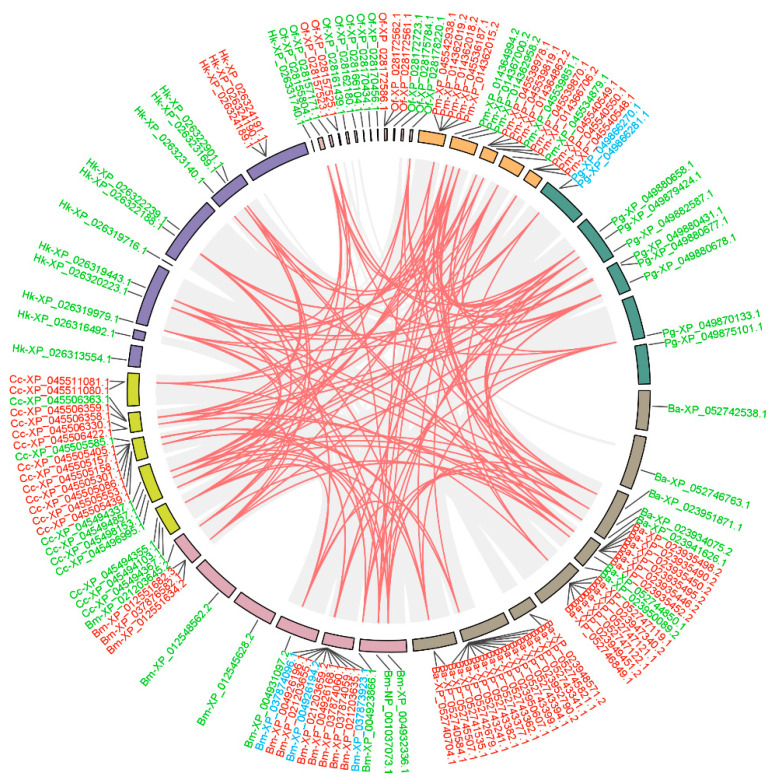
Genomic locations and duplication patterns of *GH1* genes in seven Lepidopteran species. The gene names are composed of a two-letter species abbreviation and the gene ID. Red, blue, and green represent tandem duplication, proximal duplication, and dispersed duplication, respectively. Chromosome karyotypes of different colors represent different species. The lines represent the collinear regions between contigs or scaffolds; the red lines represent the collinear regions containing the *GH1* genes. Bm, *Bombyx mori*; Hk, *Hyposmocoma kahamanoa*; Of, *Ostrinia furnacalis*; Pg, *Pectinophora gossypiella*; Ba, *Bicyclus anynana*; Pm, *Papilio machaon*; Cc, *Colias croceus*. The tandem duplications are arranged one after another on the same chromosome. The proximal duplications are located relatively close to each other on the same chromosome but not immediately adjacent. The dispersed duplication is inserted into a non-adjacent location on the same chromosome or a different chromosome, which is caused by transposition or chromosomal rearrangements.

To investigate the evolutionary dynamics of *GH1* genes, we analyzed duplication modes and collinearity across seven representative species. The majority of *GH1* genes in these species originated from tandem duplications and dispersed duplications, with only a small number of proximal duplications identified in *B. mori* and *Pectinophora gossypiella*. Most *GH1* genes were located in conserved syntenic regions shared among these taxa (Figure 4), except for minor dispersed duplications in *B. mori*, *P. gossypiella*, and *Colias croceu*, as well as limited dispersed or tandem duplications in *Hyposmocoma kahamanoa*, *Ostrinia furnacalis*, and *Bicyclus anynana*. Furthermore, syntenic *GH1* genes within the same genomic block were predominantly tandem duplicates, while dispersed duplicates were largely distributed across non-collinear regions.

### 3.4. Expression of GH1 Genes in the Developmental Stages and Tissues of B. mori

From the perspectives of developmental stages and tissue distribution, *GH1* genes exhibited the highest expression levels during the larval stage, followed by the pupal stage, with the lowest expression observed in the adult stage. Tissue-specific analysis revealed that *GH1* expression ranked highest in the testis, followed by relatively high expression in the midgut and fat body, while the posterior silk gland showed the lowest expression levels.

According to the analysis in the previous section, the *GH1* genes in *B. mori* are distributed among most phylogenetic groups except Groups F and M. Distinct gene duplication patterns are evident across these groups, correlating with divergent expression profiles. The *GH1* genes resulting from tandem duplication in Group A exhibit high expression in the midgut. Notably, Bm-XP_037876582.1 is specifically expressed in the midgut across different developmental stages, while Bm-XP_012551682.3 shows relatively high expression in the larval midgut. The other Group A *GH1* gene, generated by dispersed duplication, is specifically expressed in the larval midgut and the testes across various developmental stages of *B. mori*. Bm-XP_021203645.2 from Group C and Bm-XP_004932336.1 from Group I displayed consistently high expression in all the analyzed tissues and developmental stages. In Group J, Bm-XP_004931097.2 shows elevated expression in the trachea, epidermis, anterior silk gland, head, thorax, legs, antennae, and wings of *B. mori*. In contrast, the genes generated through tandem duplication in Group K are predominantly expressed in the midgut and hemolymph during the larval stage. Furthermore, Group T comprises approximately half of the *GH1* genes, primarily arising from tandem duplications and a few from proximal duplications, which demonstrate certain stage- and tissue-specific expression patterns. For instance, Bm-XP_004926168.1 is highly expressed in the larval midgut, while it is lowly expressed in the testes and ovaries. Bm-XP_004926196.1 shows relatively high expression in the fat body, epidermis, ovary, and head across developmental stages, as well as in the thorax, legs, antennae, and wings of adults. Similarly, Bm-XP_0221203635.1 shows high expression in the larval Malpighian tubules, while Bm-XP_037873923.1 displays elevated expression in the larval midgut and Malpighian tubules. Furthermore, Bm-XP_004926194.2 is highly expressed in the silk gland and ovary of *B. mori*. The *GH1* genes from Group S and O derived from dispersed duplication are highly expressed across most of the tissues except the hemolymph and silk gland.

## 4. Discussion

The present study provides a comprehensive understanding of glycoside hydrolase family 1 (GH1) evolution in Lepidoptera and reveals how dynamic changes in this gene family align with dietary ecology and coevolutionary pressures. In this study, the evolutionary history of the *GH1* gene family in Lepidoptera was systematically deciphered by integrating genomic data across 61 species. The number of *GH1* genes in Lepidoptera is relatively conserved, and species with closer genetic relationships have similar numbers of *GH1* genes. However, significant differences were observed across superfamilies, which may correlate with their feeding strategies and dietary preferences. Superfamilies such as Papilionoidea harbored higher *GH1* gene numbers, likely reflecting adaptations to cellulose-rich foliage or chemical defenses in host plants. For instance, Pieridae larvae predominantly feed on Brassicaceae plants that produce glucosinolate-potent defensive compounds, which are hydrolyzed by myrosinase to toxic metabolites like isothiocyanates (ITCs), nitriles, and thiocyanates upon tissue damage. These compounds inhibit larval growth or directly kill insects [34]. On the contrary, several insects such as *Pieris rapae* developed a particular detoxification mechanism utilizing GH1 enzymes to hydrolyze glucosinolate thioglucosidic bonds, converting toxic precursors into less harmful nitriles [8]. This allows them to circumvent plant myrosinase-based defenses and feed on Brassicaceae plants. In contrast, certain superfamilies, such as Cossoidea, Sesioidea, and Copromorphoidea possess fewer *GH1* gene numbers; their larvae typically feed on low-fiber or minimally defended plant tissues. For example, *Carposina sasakii* preferentially bores into young or early-maturing fruits (e.g., apples, peaches), which are soft, low in cellulose, and deficient in chemical defenses like tannins in mature fruits [35]. These *GH1* copy number differences across lineages reflect adaptations to ecological niches, particularly in digesting and detoxifying dietary components, which have driven the *GH1* gene family evolution.

This adaption may be linked to the frequent gene duplication and loss events within the *GH1* gene family in Lepidoptera. These dynamic changes in the *GH1* gene repertoire suggest a significant role for this gene family in mediating the complex interactions between Lepidopteran insects and their host plants [26]. For instance, phylogenetic analysis reveals that butterflies possess significantly higher numbers of *GH1* gene copies compared to moths (Figure 3), a divergence that may correlate with differences in dietary specialization.

Lepidoptera–plant interactions are often described as an evolutionary arms race. The ancestral Lepidoptera fed on non-vascular land plants [26], relying on plant tissues with low fiber content and minimal secondary metabolites. The emergence of angiosperms [36] and their diversified chemical defenses (e.g., alkaloids, terpenoids) [37,38] and structural adaptations (e.g., lignified cell walls) [39] drove the differentiation of feeding strategies in Lepidoptera. Most butterfly larvae (e.g., Hesperiidae) evolved highly specialized diets, often restricted to specific plant families or genera [40], whereas many moth larvae (e.g., Erebidae) retained generalist feeding habits, displaying diverse dietary patterns. Building on these observations, we propose that butterflies may leverage *GH1* gene duplications to drive functional innovations such as the specialized cleavage of host plant toxins, enabling adaptation to highly defended, narrow ecological niches. In contrast, moths, which often exhibit broader feeding preferences or primarily consume low-cellulose plants, may rely on generalized detoxification systems (e.g., cytochrome P450 enzymes, carboxylesterases) [41] rather than GH1-mediated specificity, reducing their dependence on GH1 for detoxification and facilitating adaptation to diverse, low-defense environments. These findings collectively highlight the pivotal role of the *GH1* gene family in the adaptive evolution of Lepidoptera. The colinear block localization of most *GH1* members (Figure 4) and their predominant tandem duplication patterns further establish a molecular evolutionary framework underpinning plant–insect interactions.

**Figure 5:**
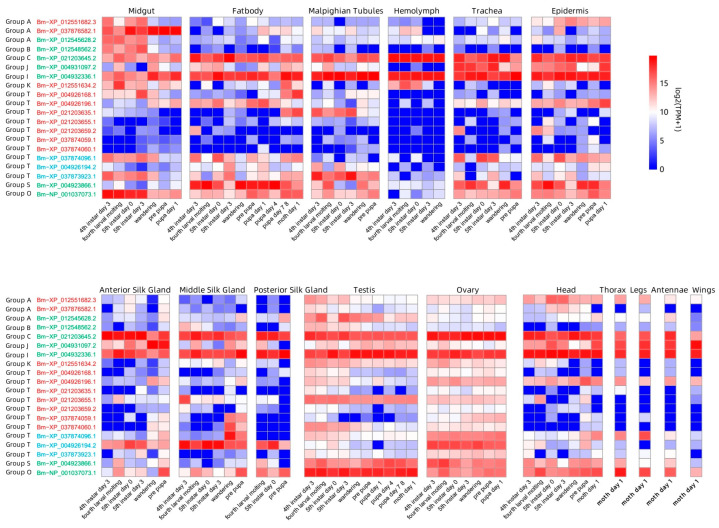
The heatmap of *GH1* gene expression across various tissues in silkworm. The *GH1* gene names of the silkworm are composed of the abbreviation of *B. mori* and the gene IDs. Red, blue, and green represent tandem duplication, proximal duplication, and dispersed duplication, respectively.

The *GH1* genes in Lepidopteran insects are mainly amplified through tandem duplication (Figure 4). Such duplicated genes usually exhibit high expression activity [42], which enhances the expression efficiency of the gene family. Lepidopterans achieve the spatiotemporal specificity of their physiological functions by regulating the expression of the *GH1* gene family. The heatmap of the expression of *GH1* genes in the silkworm (*B. mori*) shows that there is a certain specificity in tissue expression. The relatively high expression in the midgut and fat body (Figure 5) suggests that it may be involved in nutrient absorption in the midgut and energy metabolism in the fat body [43,44], while the high-level expression in the testis may be related to the morphological changes in the testis during the metamorphosis period and may also be related to the development of the testis and the formation of sperm [45]. In terms of the developmental timeline, the expression abundance in the larval stage is significantly higher than that in the pupal and adult stages, which may be directly related to the rapid growth in the larval stage and the demand for active feeding on plant materials [45]. It is worth noting that the specific expression of the genes classified as Groups B, C, and D in the larval midgut suggests that these groups may be mainly involved in digestion. Intriguingly, the silkworm (*B. mori*) lacks representatives of several clades (Groups A, F, and I). The absence of these *GH1* genes in the silkworm suggests gene loss events, potentially due to its specialized diet, lacking the specific substrates or toxins that these enzymes would otherwise act upon. Gene loss can be an adaptive outcome when a function becomes superfluous; maintaining an unnecessary enzyme carries a metabolic cost and risk of deleterious mutations, so redundant *GH1* copies may be purged over time in certain hosts [46]. On the other hand, other Lepidoptera lineages retain and even expand particular *GH1* clades through gene duplication, implying the formation of new classifications due to the emergence of new functions [47].

This pattern highlights how gene duplication and loss shape lineage-specific adaptation; certain *GH1* clades have diversified insect groups to address specific ecological challenges, while other insects have streamlined their *GH1* repertoire to align with a more specialized host range. This dynamic interplay suggests that the co-evolution of gene duplication and expression regulation in the *GH1* family plays a crucial role in enabling Lepidoptera to adapt to varying physiological demands and environmental conditions. Further research at the gene expression level will help to reveal the role of the *GH1* gene family in the adaptation process of Lepidoptera insects.

In conclusion, the *GH1* gene family plays a crucial role in the complex and dynamic interactions between insects and plants. It provides an adaptive toolkit in the Lepidopteran herbivory. The extensive distribution, diverse functions, and variation in *GH1* gene copy numbers across different Lepidopteran superfamilies reflect the adaptive strategies and evolutionary trajectories these insects have developed during their interactions with plants. The expansion, diversification, and selective loss of the *GH1* genes have enabled Lepidopteran insects to enhance both their digestive efficiency and overcome plant defenses. By integrating comparative genomics, phylogenetics, and gene expression data, our study provides a framework for understanding how gene family dynamics contribute to ecological diversification. The evolutionary dynamics of the *GH1* family in Lepidoptera exemplify how molecular evolution underpins ecological interactions, offering a deeper understanding of how insects have become one of the most successful herbivorous groups on the planet.

## Data Availability

The original contributions presented in this study are included in the article. Further inquiries can be directed to the corresponding author.
